# Thyroid Storm Presenting as Psychosis

**DOI:** 10.1177/2324709618777014

**Published:** 2018-05-18

**Authors:** Dimpi Desai, Sara Zahedpour Anaraki, Neetha Reddy, Eric Epstein, Vafa Tabatabaie

**Affiliations:** 1 Jacobi Medical Center, Albert Einstein College of Medicine, Bronx, NY, USA; 2 Montefiore Medical Center, Albert Einstein College of Medicine, Bronx, NY, USA

**Keywords:** thyroid storm, psychosis, hyperthyroidism, thyrotoxicosis, plasmapheresis

## Abstract

Thyroid storm is a life-threatening endocrine emergency with an incidence rate of 1% to 2%. It is a systemic condition of excessive thyroid hormone production and release leading to thermoregulatory, adrenergic, neuropsychiatric, cardiovascular, and abdominal manifestations. Although it is a rare condition, it carries a significant mortality rate. Hence, knowing the common and uncommon presentations of thyroid storm is important for its prompt diagnosis and treatment. In this article, we present an unusual case of a young woman who presented with psychosis as the manifesting symptom of thyroid storm. She did not respond adequately to conventional medical treatment, requiring plasmapheresis and a definitive thyroidectomy, which ultimately led to the return of patient’s baseline mental status and a dramatic recovery.

## Case Presentation

A 28-year-old Hispanic female with a past medical history of migraine, depression, and a family history of schizophrenia was brought to the emergency room by her family as she was not acting her usual self and was demonstrating strange behavior for 1 week prior to admission. The patient’s family described new onset, and rapidly worsening paranoid delusions along with auditory and visual hallucinations. Associated symptoms included nausea, vomiting, and diarrhea. She denied taking any medications or supplements. Vital signs at the time of admission showed temperature of 99.6°F, heart rate of 144 beats per minute, and elevated blood pressure of 160/72 mm Hg. Physical examination was notable for an enlarged and smooth thyroid gland with an audible bruit. At the time of initial interview, she was emotional and tearful, talking and smiling inappropriately, and admitted to seeing people in the room who were not present.

## Investigations

Laboratory investigations showed severe hyperthyroidism with suppressed thyroid-stimulating hormone (TSH), elevated free and total thyroxine hormone (T4), elevated total triiodothyronine (T3), positive antithyroid peroxidase antibodies, and thyroid stimulating immunoglobulin (Table 1). Liver transaminases were mildly elevated (alanine transaminase 84 and aspartate transaminase 64). Electrocardiogram demonstrated sinus tachycardia. Urine toxicology screen, computed tomography scan of the head, and infectious workup were negative. Given her altered mental status (20 points), tachycardia (25 points), abdominal symptoms (10 points), and fever (5 points), a diagnosis of thyroid storm was made, with a Burch and Wartofsky score of 60 (Table 2). Thyroid ultrasound showed an enlarged, heterogeneous hyperemic gland.

**Table 1.** Laboratory Parameters of the Case.

Thyroid Function Tests With Reference Ranges	On the Day of Admission	10 Days After Receiving Standard Antithyroid Treatment	Following 2 Sessions of Plasmapheresis	Post-Thyroidectomy
TSH (0.4-4.6 µU/mL)	<0.005	<0.005	0.011	0.006
Thyroxine, T4 (5-12 µg/dL)	20.48	21.7	7.79	7.96
Free T4 (0.8-1.7 ng/dL)	>7.77	—	3.63	2.59
Total T3 (81-199 ng/dL)	403	513	—	81
Thyroid stimulating immunoglobulin (<140%)	514		—	—
Thyroid peroxidase antibody (0.0-5.0 IU/mL)	659.7			

Abbreviations: TSH, thyroid stimulating hormone; T4, free and total thyroxine hormone; T3, total triiodothyronine.

**Table 2.** Diagnostic Criteria of Thyroid Storm (Burch and Wartofsky Scoring)^a^.

Diagnostic Criteria	Scoring Points
Thermoregulatory dysfunction	
Temperature	
99-99.9°F	5
100-100.9°F	10
101-101.9°F	15
102-102.9°F	20
103-103.9°F	25
>104°F	30
Central nervous system effects	
Absent	0
Mild (agitation)	10
Moderate (delirium, psychosis, and extreme lethargy)	20
Severe (seizures and coma)	30
Gastrointestinal-hepatic dysfunction	
Absent	0
Moderate (diarrhea, nausea/vomiting, and abdominal pain)	10
Severe (unexplained jaundice)	20
Cardiovascular dysfunction	
Tachycardia (beats per minute)	
90-109	5
100-119	10
120-129	15
>140	25
Congestive heart failure	
Absent	0
Mild (pedal edema)	5
Moderate (bibasilar rales)	10
Severe (pulmonary edema)	15
Atrial fibrillation	
Absent	0
Present	10
Precipitating event	
Absent	0
Present	10

a Adapted from Burch and Wartofsky.^1^

## Treatment

Treatment for thyroid storm was immediately started including propranolol 60 mg 3 times daily, methimazole 20 mg twice daily, and saturated solution of potassium iodide 5 drops every 6 hours, given an hour after first dose of methimazole. Propylthiouracil was avoided due to patient’s elevated transaminases. Beta-blockers were titrated up to control her heart rate, her vital signs improved and her abdominal symptoms resolved. However, despite continuing methimazole and saturated solution of potassium iodide, T3, and T4 levels continued to rise (Table 1) and psychosis with agitation, delusions, and hallucinations persisted. Hydrocortisone was added at this time to reduce the peripheral conversion of T4 to T3; however, she continued to fail to respond to standard medical therapy. In view of worsening thyroid function tests and worsening psychosis despite 10 days of treatment, and due to the concern for an impending decompensation due to thyroid storm, a decision was made to proceed with plasmapheresis in preparation for an urgent thyroidectomy. She received 2 rounds of plasmapheresis, which resulted in normalization of thyroid hormone levels followed by total thyroidectomy. The postsurgical course was uncomplicated; she was successfully extubated, her mental status returned to baseline, and she was discharged several days later on thyroid hormone replacement. After functioning normally for a month, she returned to the emergency room for psychotic behavior, at which time diagnosis of schizophrenia was made and she was started on olanzapine. Her free T4 level was normal at this time.

## Review of Literature

Thyroid storm is a rare, but life-threatening complication of hyperthyroidism.^1,2^ The most common etiology of thyroid storm is Graves’ disease, as was seen in our patient, followed by other causes of hyperthyroidism such as a solitary toxic adenoma or toxic multinodular goiter.^3^ Rare causes of hyperthyroidism such as hypersecretory thyroid carcinoma, struma ovarii, thyrotropin-secreting pituitary tumor, and administration of iodine via radiocontrast dye or amiodarone are also among reported etiologies.^4^ A precipitating event is usually identified that results in transition from hyperthyroid state to thyroid storm. In the past, thyroid surgery during uncontrolled hyperthyroidism was the most common reason for thyroid storm; presently, infection is the most common precipitant. Other precipitating factors include myocardial infarction, pulmonary thromboembolism, parturition, surgery, trauma, diabetic ketoacidosis, withdrawal of antithyroid drugs, and administration of iodine (intravenous radiocontrast dye or amiodarone).^5^ We were not able to identify a precipitating cause in our patient. However, her strong family history of schizophrenia might have made her susceptible to developing psychosis.

Although the diagnosis of thyroid storm is based on clinical presentation, laboratory values of elevated free T3 and free T4 with a suppressed TSH are invariably present. Since the clinical symptoms are vague and involve multiple systems, Burch and Wartofsky have created a point system to standardize and help make an objective diagnosis of thyroid storm (Table 2). A score of 45 or above is highly suggestive of thyroid storm, a score of 25 to 44 suggests an impending storm, and a score less than 25 is unlikely of being thyroid storm. Our patient had a score of 60.^1^ Our patient also met the criteria of thyroid storm (TS1) using the Japanese Thyroid Association criteria as she had elevated thyroid hormone levels along with central nervous system (CNS) manifestation, tachycardia, gastrointestinal symptoms, and fever.^6^

Four main clinical features of thyroid storm include fever, tachycardia, gastrointestinal symptoms, and CNS involvement.^7^ CNS manifestations of thyrotoxicosis fall in a vast spectrum ranging from emotional lability, anxiety, agitation, confusion, delirium, paranoia, mania, frank psychosis, seizures, and even coma.^4^ The association between psychosis and thyrotoxicosis has been suggested in a review of 18 patients with acute psychosis and newly diagnosed thyrotoxicosis.^8^ In a recent case series of 28 patients with thyroid storm, 8 of them were seen to have neuropsychiatric involvement.^9^ Although there are individual case reports of Graves’ disease and thyrotoxicosis presenting as psychosis,^10-14^ there are only a handful of cases reported in the literature where psychosis was the presenting feature of thyroid storm. Chen et al^15^ described the case of a 55-year-old male who presented with hallucinations and delusions refractory to psychiatric medications, diagnosed with thyroid storm on the fourth day of admission, with a dramatic improvement in psychiatric symptoms after receiving antithyroid treatment. Lu et al^16^ described the case of a 14-year-old presenting with odd behavior who was diagnosed with thyroid storm after suffering an episode of seizure on the second day of admission, with prompt resolution of symptoms after receiving standard antithyroid treatment.^16^

A number of theories attempt to explain the pathogenesis of hyperthyroidism causing neuropsychiatric symptoms. Hyperthyroidism modulates β-receptor density in the brain as well as their sensitivity to catecholamines; this augmentation of β-receptor-mediated adrenergic activity is thought to be responsible for neuropsychiatric symptoms.^17,18^ TSH receptors are largely present in the hippocampus and cortex of the brain. Their increased stimulation by TSH receptor antibodies in Graves’ disease, leading to an excessive local production of T3 is also thought to contribute to psychiatric symptoms.^19^ It has been demonstrated that thyroid hormones increase Na^+^ current density in the hippocampal region in rat models, thus causing neuronal excitability that could explain neurologic symptoms.^20^ Using positron emission tomography, Schreckenberger et al^21^ demonstrated altered glucose metabolism in the limbic structures of the brains of patients with untreated Graves’ hyperthyroidism. A study by Miao et al^22^ further showed that these changes in cerebral glucose metabolism seen in hyperthyroid states correct after treatment. These findings suggest that abnormalities in thyroid function can result in changes in neurochemistry and cerebral metabolism. Involvement of limbic structures can result in psychiatric manifestations seen in hyperthyroidism.

In addition to the unusual manifestation of thyroid storm, our patient’s resistance to the conventional medical management with worsening psychosis and hyperthyroidism added to the challenge of management. There are very few studies in literature describing cases and etiologies of resistant thyrotoxicosis. Refractory cases of thyroid storm have been reported either as results of amiodarone-induced thyrotoxicosis^23^ or Grave’s disease.^24-27^ These cases have been refractory to thionamides, β-blockers, and, rarely, to iodine as well. The possible mechanisms of resistance to methimazole include malabsorption, accelerated drug metabolism, presence of antibodies binding to methimazole or impaired intrathyroidal accumulation, and action of methimazole.^27^ Dahlberg et al^28^ have suggested that failure of methimazole therapy is not primarily due to aberrations in drug metabolism, but related to the severity of the disease, especially elevated T3 levels. It has also been postulated that iodine interferes with the response of thyroid to antithyroid drugs indirectly by increasing thyroidal stores of preformed hormone and directly by altering carbimazole’s metabolism in the thyroid.^29^ Further studies need to be done to establish more clearly why certain cases like ours are resistant to conventional medical management.

The standard medical management of thyroid storm requires a multidrug approach in order to block the production (thionamides), release (inorganic iodine), and peripheral effects of thyroid hormones (β-blockers and steroids). Only exceptional cases are refractory to this conventional treatment and need other methods to remove excess thyroid hormones. Plasmapheresis, peritoneal dialysis, charcoal hemoperfusion, and resin hemoperfusion have shown to rapidly remove excess thyroid hormones in thyroid storm.^30-34^ During plasmapheresis, patient’s plasma is extracted from other blood components and a replacement colloid solution such as 5% albumin is infused back, thus removing thyroid binding globulin along with bound hormones. The replacement solution such as albumin provides new binding sites for free thyroid hormones present in the circulation and thus leads to reduction of free thyroid hormone levels.^35^ Since thyroid surgery can be associated with significant perioperative mortality in uncontrolled thyrotoxicosis, plasmapheresis is an effective therapeutic procedure that helps achieve a euthyroid state before a definitive thyroidectomy.

Standard antithyroid therapy along with β-blockers remains the first line of treatment for psychiatric symptoms in thyroid storm. Adjunctive treatment with antipsychotics is warranted if psychosis persists. Whereas some studies have reported complete resolution of psychiatric symptoms after hyperthyroidism was treated,^36,37^ several studies have described the persistence of mental symptoms even after achieving a euthyroid state.^38^

Thus, we present a case of thyroid storm with 2 unusual characteristics, psychosis being the initial manifesting feature of thyroid storm and its refractory nature to standard medical treatment, requiring plasmapheresis and eventual thyroidectomy.

## Key Learning Points

1. Neuropsychiatric symptoms including frank psychosis could be a presenting feature of thyroid storm.
2. When a patient presents with psychosis, thyroid storm must be considered in the differential diagnosis in the appropriate clinical scenario and prompt treatment be started in cases of high suspicion.
3. Close clinical monitoring, usually in intensive care unit, is required in thyroid storm.
4. For cases refractory to standard treatment, plasmapheresis can be done for removal of excess thyroid hormones and help achieve a euthyroid state before definitive thyroidectomy can be performed.
